# Binary Tönnis classification: simplified modification demonstrates better inter- and intra-observer reliability as well as agreement in surgical management of hip pathology

**DOI:** 10.1186/s12891-020-03520-x

**Published:** 2020-07-29

**Authors:** Jacob Shapira, Jeffrey W. Chen, Rishika Bheem, Philip J. Rosinsky, David R. Maldonado, Ajay C. Lall, Benjamin G. Domb

**Affiliations:** 1 American Hip Institute Research Foundation, Des Plaines, IL 60018 USA; 2 Vanderbilt University School of Medicine, Nashville, TN 37232 USA; 3 American Hip Institute, 999 E Touhy Ave, Suite 450, Des Plaines, IL 60018 USA; 4 AMITA Health St. Alexius Medical Center, Hoffman Estates, IL 60169 USA

**Keywords:** Tönnis classification, Hip osteoarthritis, Total hip Arthroplasty, Hip arthroscopy, Hip Arthroplasty

## Abstract

**Background:**

The traditional Tönnis Classification System has inherent drawbacks as it is vulnerable to the subjectivity of a four-grade system. A two-grade classification could potentially be more reliable. The purpose of this study is to (1) compare the inter-observer and intra-observer reliability of the traditional Tönnis Classification System and a simplified Binary Tönnis Classification System for hip osteoarthritis and to (2) evaluate the clinical applicability of both systems. Our hypothesis is that the proposed Binary Tönnis Classification System will have better reliability and agreement for surgical decision-making.

**Methods:**

Forty consecutive patients were selected to participate in this study. Patients were included in this study if they were between 35 and 60 years old. Patients were excluded if they had prior hip surgeries or conditions. All radiographs were randomized and blinded by a non-observer. Five fellowship-trained hip surgeons from a single center, in a fully crossed design, analyzed and graded all the radiographs utilizing the traditional Tönnis Classification System and the proposed Binary Tönnis Classification System. Intra- and inter-observer reliability values for both the systems were calculated using the Cohen’s κ coefficient. A multi-rater κ was calculated using the weighted Fleiss method.

**Results:**

The study sample contained 40 anterosuperior hip radiographs. For the traditional Tönnis Classification System, the weighted κ showed a fair inter-observer reliability (κ = 0.474) and excellent intra-observer reliability (κ mean = 0.866). For the proposed Binary Tönnis Classification System, both inter-observer and intra-observer reliability demonstrated excellent values, (κ = 0.858 and 0.928, respectively). On average, the Binary Tönnis Classification System correctly captured 87% of cases. When the traditional Tönnis Classification System was dichotomized, the capture rate was 84%.

**Conclusion:**

A simplified binary Tönnis Classification System demonstrates better reliability and clinical implementation than the traditional Tönnis Classification System.

## Background

For hip joint pathologies, two major operative treatments exist: hip preservation and hip replacement. The presence of osteoarthritis is a critical factor in a surgeon’s decision between the two options [1]. Efforts to preserve the hip joint are hindered by the presence of osteoarthritis [2]. Therefore, a reliable evaluation of the degree of osteoarthritis is necessary for optimizing patient outcomes. Radiographic assessment provides essential information concerning the diagnosis and treatment of osteoarthritis [3]. The traditional Tönnis Classification System is commonly used to classify the severity of osteoarthritis. The literature generally supports hip preservation for hips graded as Tönnis 0 and 1, and replacement for hips graded Tönnis 2 and 3 [2, 4]. However, despite its extensive use in clinical practice and medical literature, the traditional Tönnis Classification System has some drawbacks [5]. First, several studies have reported questionable inter-observer and intra-observer reliability [3, 6, 7]. A cardinal drawback of the traditional Tönnis Classification System is it’s subjectivity. It has been criticized for being unclear and having overlapping parameters. Yet, another difficulty may rise when parameters from different grades are found in a single radiograph e.g. moderate loss of head sphericity and slight narrowing of the joint space, which pretrain to grade 2 and 1, respectively [5]. The pitfalls associated with the traditional Tönnis Classification System reach beyond the boundaries of orthopedics and may have multidisciplinary manifestations that impair the cross talk between radiologists, general practitioners, and rheumatologists. Similar to the traditional Tönnis Classification System, the Garden Classification for femoral neck fractures also demonstrated poor reliability derived from the challenging radiographic distinctions between the grades. Based on the clinical relevancy of the Garden Classification, a simplified binary classification was developed that demonstrated higher reliability compared to the original classification [8–11]. Given the binary nature of available surgical interventions (i.e. hip preservation versus reconstruction) derived from the traditional Tönnis Classification System, a two-level classification could be more reliable and reproducible without compromising the clinical relevance. Taking into consideration Occam’s Razor [12], which states that the simplest answer is typically the correct answer, a two-level classification for surgical treatment options seems most appropriate. The goal of this study is to validate a simplified Binary Tönnis Classification System to reduce excessive complexity and better capture the diagnostic essence of having a certain classification. Specifically, this study (1) compares the inter-observer and intra-observer reliability of the traditional Tönnis Classification System and a new simplified Binary Tönnis Classification System for hip osteoarthritis and (2) evaluates the clinical applicability of both systems, notably its agreement with the clinician’s decision for either preservation or replacement. Our hypothesis is that a binary system will have better reliability and agreement for surgical decision-making.

## Methods

### Patient selection and data acquisition

Forty consecutive patients who presented to the clinic for hip pain between February 2018 to March 2018 were selected to participate in this study. Patients were included in the study if they were between the ages of 35 and 60 years old. Patients were excluded if they had prior ipsilateral or contralateral surgeries or had prior hip conditions such as Legg-Calve-Perthes disease, slipped capital femoral epiphysis, pigmented villonodular synovitis, or ankylosing spondylitis. All patients underwent operative management due to radiographic FAI, osteoarthritis, and/or symptoms of hip pain that were unresponsive to conservative treatment and significantly limited activities. Demographic data, such as sex of patients, laterality, and age at surgery, was collected for all patients.

All patients underwent routine radiographic imaging at their preoperative clinic visit. A standard anteroposterior supine radiograph was used for this study to grade the severity of osteoarthritis, the protocol for which is detailed by Clohisy et al. [13]

This study was approved by the Institutional Review Board and did not receive any funding. All patients participated in the American Hip Institute Hip Preservation Registry through written consent. While the present study represents a unique analysis, data on some patients in this study may have been reported in other studies.

### Classification systems

The traditional Tönnis Classification (Table 1) and the simplified Binary Tönnis Classification systems (Table 2**)** were used in this study. The simplified Binary Tönnis Classification System was fashioned to reflect the primary indications that our institution uses with the traditional Tönnis Classification System: hip preservation or reconstruction.

**Table 1:** Traditional Tönnis Classification

Tönnis Grade	Description
Grade 0	- No signs of osteoarthritis
Grade I	- Sclerosis of the joint with minimal joint space narrowing and osteophyte formation
Grade II	- Small cysts development with moderate joint space narrowing
Grade III	- Advanced arthritis with large cysts, joint space - Joint space obliteration, severe deformation of the femoral head

**Table 2:** Simplified Binary Tönnis Classification

Classification	Description
Grade 0	- No or minimal joint space narrowing - No or slight osteophytes - No or slight sclerosis
Grade I	- Joint space narrowing - Loss of head sphericity - Cysts development - Avascular necrosis

### Inter-rater reliability and agreement with surgical treatment

**Fig. 1:**
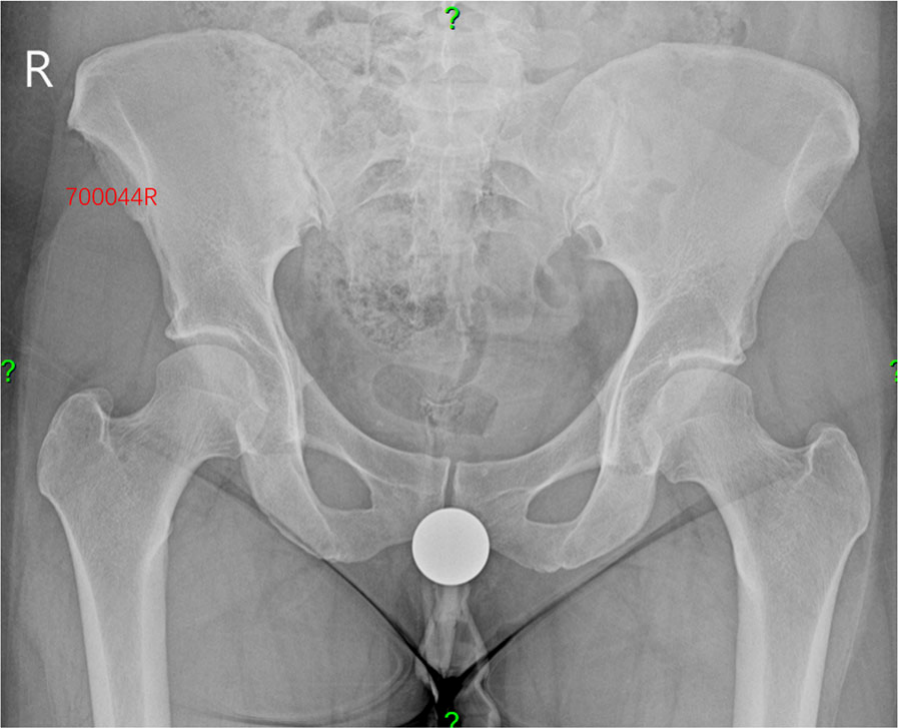
Image of blinded hip radiograph. The medical reference number and the side under consideration is in red

Five fellowship-trained hip surgeons from a single center were the observers for this study. Three observers were hip preservation and reconstruction fellows and two observers were attendings who had trained in both hip preservation and reconstruction. Radiographic grading of hip OA is part of the observers’ daily practice. However, to minimize inter-observer discrepancies, both the traditional Tönnis Classification System and the Binary Tönnis Classification System were provided on each individual excel sheet that was utilized to grade the radiographs. This study was a full-crossed study in which all observers read the same set of radiographs. All images were uploaded to the digital imaging system and retrieved by a non-observer who randomized and blinded the films, Fig. 1.

The five observers independently assessed the series of radiographs. Observers classified the radiographs utilizing the traditional Tönnis Classification System and rated another set of randomized radiographs with the Binary Tönnis Classification System after at least a week had transpired. Images were randomized again, and observers repeated their respective assessment at least 3 weeks later.

### Statistical analysis

Statistical analysis was conducted in R (R software foundation, version 3.6.0) and Microsoft Excel (Redmond, WA). Demographic data was separated and analyzed for patients who underwent arthroscopy or THA. To analyze demographic data, the Chi-squared and Fisher’s Exact tests were utilized to evaluate differences in the proportions of categorical data between the arthroscopy and THA groups. For continuous variables, the F-test was performed to evaluate variance, and the Shapiro-Wilk test was utilized to evaluate distribution. A *p* > 0.05 indicated equal variance and normal distribution, respectively. The independent-samples t-test was performed for unpaired data comparisons between both groups. Significance was set to 0.05.

Intra-observer and inter-observer reliability were calculated using the Cohen’s κ coefficient for the traditional Tönnis Classification System and the simplified Binary Tönnis Classification System. Further, the traditional Tönnis Classification System was dichotomized (0 and 1 vs. 2 and 3) and the Cohen’s κ coefficient was calculated. The multi-rater κ was calculated using the weighted Fleiss method. The degree of agreement based on the κ coefficient were interpreted by the ranges recommended by Landis and Koch: a κ value of 0–0.2 indicated slight agreement, 0.2–0.4 to be fair, 0.4–0.6 to be moderate, 0.6–0.8 to be substantial, and greater than 0.8 to be near perfect [14].

The traditional Tönnis Classification System and the simplified Binary Tönnis Classification System were assessed for agreement with the surgical treatment received by the patient (either hip preservation or hip replacement).

## Results

The study sample contained 40 anterosuperior hip radiographs, 19 of which received hip preservation and 21 of which received hip replacement. There were 15 males and 25 females (age 35.05–59.25 years). The demographics of the overall group and subgroups are presented in Table 3.

**Table 3:** Demographics

	All	Scope	Arthroplasty	*P*-Value
Gender (Male: Female)	(15:25)	(3:16)	(12:9)	0.006
Side (R:L)	(21:19)	(10:9)	(11:10)	0.98
Age, years mean (sd, range)	47.74 (7.44,35.05, 59.26)	43.58 (6.47,35.05, 54.03)	51.68 (6.86,35.81, 59.26)	< 0.001

The traditional Tönnis Classification System showed fair reliability for the inter-observer reliability, (κ = 0.474) and excellent reliability for the intra-observer reliability (κ_mean_ = 0.866, range = 0.780–0.907), as calculated by the weighted κ agreement.

The inter-observer and intra-observer reliability showed improvement with the simplified Binary Tönnis Classification System. The inter-observer reliability was (κ = 0.858) and intra-observer reliability was (κ _mean_ = 0.928, range = 0.892–0.948). Both inter-observer and intra-observer reliability were deemed excellent **(**Table 4**).**

**Table 4:** Intra-observer reliability of the classification systems

	Observer 1	Observer 2	Observer 3	Observer 4	Observer 5
Traditional Tönnis Classification System	κ = 0.883	κ = 0.578	κ = 0.780	κ = 0.884	κ = 0.907
Dichotomized Tönnis Classification System	κ = 0.949	κ = 0.948	κ = 0.521	κ = 0.848	κ = 0.948
Binary Tönnis Classification System	κ = 0.948	κ = 0.948	κ = 0.9	κ = 0.948	κ = 0.892

The Tönnis grading based on both systems and their agreement with the ultimate surgical management were calculated and are represented in Table 5. On average, the simplified Binary Tönnis Classification System correctly captured 87% of cases. When the traditional Tönnis Classification System was dichotomized (0 and 1 as hip preservation and 2 and 3 as hip replacement), the capture rate was 84%. The confusion matrices for the capture rates are depicted in Tables 6 and 7.

**Table 5:** Agreement between assessed parameters on plain x-ray

		Reader 1	Reader 2	Reader 3	Reader 4	Reader 5	Average
Tönnis Classification	0	18 (45%)	17 (42.5%)	2 (5%)	10 (25%)	13 (32.5%)	12 (30%)
	1	5 (12.5%)	7 (17.5%)	10 (25%)	12 (30%)	11 (27.5%)	9 (22.5%)
	2	3 (7.5%)	5 (12.5%)	12 (30%)	5 (12.5%)	5 (12.5%)	6 (15%)
	3	14 (35%)	11 (27.5%)	16 (40%)	13 (32.5%)	11 (27.5%)	13 (32.5%)
Binary Classification	0	24 (60%)	23 (57.5%)	20 (50%)	24 (60%)	22 (55%)	22.6 (56.5%)
	1	16 (40%)	17 (42.5%)	20 (50%)	16 (40%)	18 (45%)	17.4 (43.5%)
Tönnis Relation to Surgery	Preservation (*n* = 19)	19 (100%)	19 (100%)	10 (52.63%)	17 (89.47%)	19 (100%)	16.8 (88.42%)
	Replacement (*n* = 21)	17 (80.95%)	16 (76.19%)	19 (90.48%)	16 (76.19%)	16 (76.19%)	16.8 (80%)
		36 (90%)	35 (87.5%)	29 (72.5%)	33 (82.5%)	35 (87.5%)	33.6 (84%)
Binary Relationship to Surgery	Preservation (*n* = 19)	19 (100%)	19 (100%)	17 (89.47%)	19 (100%)	17 (89.47%)	18.2 (45.5%)
	Replacement (*n* = 21)	16 (76.19%)	17 (80.95%)	18 (85.71%)	16 (76.19%)	16 (76.19%)	16.6 (41.5%)
		35 (87.5%)	36 (90%)	35 (87.5%)	35 (87.5%)	33 (82.5%)	34.8 (87%)

**Table 6:** Confusion Matrix for Dichotomized traditional Tönnis Classification System

Surgeon-Recommended Treatment	Observer 1		Observer 2		Observer 3		Observer 4		Observer 5	
	Preservation	Replacement	Preservation	Replacement	Preservation	Replacement	Preservation	Replacement	Preservation	Replacement
A: First Round of Reads										
Preservation	19	0	19	0	10	9	17	2	19	0
Replacement	4	17	5	16	2	19	5	16	5	16
B: Second Round of Reads										
Preservation	19	0	18	1	14	5	19	0	18	1
Replacement	3	18	5	16	3	18	4	17	5	16

**Table 7:** Confusion Matrix for Binary Tönnis Classification System

Surgeon-Recommended Treatment	Observer 1		Observer 2		Observer 3		Observer 4		Observer 5	
	Preservation	Replacement	Preservation	Replacement	Preservation	Replacement	Preservation	Replacement	Preservation	Replacement
A: First Round of Reads										
Preservation	19	0	19	0	17	2	19	0	17	2
Replacement	5	16	4	17	3	18	5	16	5	16
B: Second Round of Reads										
Preservation	19	0	19	0	16	3	19	0	16	3
Replacement	4	17	5	16	4	17	4	17	5	16

## Discussion

The aim of this study was to validate a simplified Binary Tönnis Classification System. In this study, 40 radiographs of consecutive patients were analyzed by five fellowship-trained orthopedic surgeons. Overall, the Binary Tönnis Classification System reported better inter-observer and intra-observer reliability and demonstrated higher agreement rate with the ultimate surgical treatment, as recommended by the treating surgeon, compared to the traditional Tönnis Classification System.

In their study, Clohisy et al. [3] (Table 8**)** evaluated the ability of hip specialists to reliably indicate the correct diagnosis based on plain radiographs alone. Five hip specialists and one fellow performed a blinded radiographic review of 25 hips with developmental dysplasia, 27 hips with femoroacetabular impingement, and 25 control hips. The readers assessed a variety of radiographic parameters including osteoarthritis using the traditional Tönnis Classification System. The combined κ for intra- and inter-observer reliability for the traditional Tönnis Classification System were 0.60 (95% CI: 0.54–0.66) and 0.59, respectively. Furthermore, Steppacher et al. [6] had two readers assess the Tönnis grade for a set of 50 radiographs illustrating dysplastic hips. The range of reported κ for intra-observer were 0.73 to 0.74. The Fleiss κ for interobserver reliability was 0.74. Clohisy et al. attributed the difference between their results and Steppacher’s to their inclusion of a non-dysplastic cohort, in contrast to a dysplastic only cohort in Steppacher’s study. The higher radiographic variability may have contributed to a decrease in reliability, especially in cases with none or mild arthritis. Troelsen et al. [7] aimed to investigate the variability of diagnostic assessment of the hip joint. In their study, four observers independently assessed the level of osteoarthritis in 25 radiographs. Treolsen dichotomized Tönnis grades. They assessed the dichotomized inter-observer reliability, of a quaternary classification, as well as its agreement with CT scan. The κ for inter-observer agreement was 0.54 for the Tönnis classification and 0.66 for the dichotomized version. Furthermore, the observed agreement with the CT scan was 70% for the traditional Tönnis Classification System and 88% for the dichotomized alternative. In this present study, κ for intra- and inter-observer reliability for the traditional Tönnis Classification System were 0.86 and 0.47, respectively. In contrast to the evidence reported for the traditional Tönnis Classification System, the simplified Binary Tönnis Classification System demonstrated excellent inter- and intra-observer κ (i.e. 0.86 and 0.85, respectively). Additionally, this study supports Troelsen’s findings from dichotomizing the traditional Tönnis Classification System. Adopting a true binary classification will better serve the clinician as it would eliminate the need for a preliminary low-reliable four level classification which requires further dichotomization for determining treatment.

**Table 8:** Previously published X-ray Reliability Studies for the Traditional Tönnis Classification System

Study	Observers	Radiographs	Intraobserver	Interobserver
Troelsen et al.	4	25	Grade 0–1 vs 2–3: 0.54 Grade 0–3: 0.66	
Cloohisy et al.	6	77	K = 0.60 (95% CI: 0.54–0.66)	K = 0.59
Steppacher et al.	2	75	K = 0.73 and 0.76	K = 0.74
Valera et al.	2	117	K = 0.364–0.397	K = 0.173–0.397
Hiza et al	3	49	K = 0.472	K = 0.287
Current Study	5	40	Traditional Tönnis K = 0.474 Binary Tönnis K = 0.866	Traditional Tönnis K = 0.858 Binary Tönnis K = 0.928

The second step in validating the simplified Binary Tönnis Classification System was to assess its reliability in indicating the surgeon-recommended treatment. Valera et al. [1] evaluated the reliability of the traditional Tönnis Classification System as a reference for hip preservation. Three orthopedic surgeons examined 117 hip x-rays for hip joint osteoarthritis according to the traditional Tönnis Classification System. The κ value for interobserver reliability were slight or fair (0.173–0.379) and the κ value for intra-observer reliability were fair (0.364–0.379). Variance in classifying low grade osteoarthritis was the major cause for disagreement between observers. In contrast, experience did not play a significant role in grading reproducibility. The authors concluded that the traditional Tönnis Classification System is a poor method of assessing early hip osteoarthritis and that routine use in clinical decision-making for preservative surgery should be reconsidered. In this study, the traditional Tönnis Classification System correctly overlapped with actual surgical treatment in 85.2% of cases. The simplified Binary Tönnis Classification System had a higher overlap, correctly capturing 86.5% of the cases. While the binary classification did show a slightly better correlation with the indicated treatment, it should be emphasized that radiographic evaluation is only part of the overall patient assessment and thus a discrepancy between both classification systems and the actual performed treatment should be expected.

In summary, the simplified Binary Tönnis Classification System addresses the drawbacks of the traditional Tönnis Classification System without compromising clinical relevance. Adoption of a binary system would allow for more consistent data collection, thus improving the quality of studies. Practically for the clinician, a two-grade classification is more appropriate for a two-way treatment.

### Limitations

The major limitation of this study stems from the retrospective nature. We minimized this effect by blinding the investigators to any identifier including name and treatment. In addition, we excluded patients who were treated contralaterally, which could bias grading. Also, the readers in this study were all surgeons. A better generalization may have been generated with the inclusion of multidisciplinary readers (e.g. radiologists). Furthermore, whereas the actual procedure is performed by senior surgeon, either arthroscopy or arthroplasty, was indicated based on the overall patient’s assessment, the assigned procedures in this study were exclusively based on the radiographic classifications. This single blinding design may have introduced a bias to the study. Despite the effort to minimize the selection bias in the study by choosing consecutive series of patients, the resulted cohort was fairly homogenic in terms of demographic characteristics, which by itself may limit the generalization of the results. Last, patients without osteoarthritis, who traditionally were classified as Tönnis 0, no longer have a distinct grade according to the binary classification. This may potentially lead to lower threshold for indicating surgery. However, since hip arthroscopy is generally indicated based on the intra-articular mechanical impairments such as FAI and labral tears, osteoarthritis is normally considered a contraindication for such preservative measures.

## Conclusion

A simplified Binary Tönnis Classification System demonstrates better reliability and clinical implementation than the Traditional Tönnis Classification System.

## Data Availability

The datasets generated and analyzed during the current study are not publicly available due to Health Insurance Portability and Accountability Act (HIPAA) regulations.

## Abbreviations

R: Right
L: Left
sd: standard deviation
n: sample size
CI: Confidence interval
